# Efficacy of Closed-Incision Negative Pressure Wound Therapy in Reducing Postoperative Complications in Breast Reconstruction After Radiotherapy: A Propensity Score Analysis

**DOI:** 10.1093/asjof/ojae073

**Published:** 2024-09-02

**Authors:** Khaled O Alameddine, Cristina A Salinas, Maria Yan, Jorys Martinez-Jorge, Aparna Vijayasekaran, Nho V Tran, Christin A Harless

## Abstract

**Background:**

Implant-based breast reconstruction following radiotherapy can lead to significant postoperative complications. Closed-incision negative pressure wound therapy (ciNPWT) has emerged as a potential intervention to reduce these complications.

**Objectives:**

To assess the effectiveness of ciNPWT in reducing postoperative complications in patients undergoing implant-based breast reconstruction after radiotherapy.

**Methods:**

A retrospective single-center cohort study was conducted, including patients who underwent implant-based breast reconstruction after mastectomy and radiotherapy between January 1, 2015, and December 31, 2022. We utilized a procedure-level analysis model with patients contributing distinct observations for multiple procedures. Our primary outcome measures included fluid collection, infection, and wound complications. Propensity score analysis was employed to adjust for potential confounders, such as BMI, smoking history, and diabetes history, creating a balanced comparison between the ciNPWT-treated and untreated groups.

**Results:**

In our study of 301 breast reconstructions postradiotherapy from 2015 to 2022, encompassing 218 unique patients, we found significant benefits of ciNPWT. During an average of 2.2-year follow-up, the ciNPWT group demonstrated no infections, contrasting with a 10.4% rate in the non-ciNPWT group (*P* < .0001). Wound complications were also significantly lower in the ciNPWT group (1.9% vs 11.2%; *P* = .00848). Demographic differences were adjusted using inverse probability of treatment weights. The findings suggest ciNPWT’s promising role in enhancing postoperative outcomes in breast reconstruction postradiotherapy.

**Conclusions:**

Our study suggests that the use of ciNPWT in implant-based breast reconstruction postradiotherapy can potentially reduce postoperative complications. This intervention can improve patient outcomes and may offer cost-saving benefits in the long run.

**Level of Evidence: 3 (Therapeutic):**

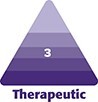

Breast reconstruction is a vital aspect of comprehensive breast cancer care, aiming not only to restore physical appearance but also to significantly improve the quality of life for survivors. Nevertheless, such procedures, particularly those following mastectomy and radiotherapy, frequently encounter significant postoperative complications.

These challenges, including impaired wound healing, seroma formation, and infections, present a considerable burden to both the patient and healthcare provider. They are particularly pronounced in the context of implant-based reconstruction and radiation therapy, thus demanding specific attention and prophylactic measures to ensure the most favorable outcomes.^1-4^

Implant-based reconstruction, despite the associated complications, continues to be favored in certain scenarios for postmastectomy irradiated patients due to its numerous advantages. These include a less invasive approach, reduced postoperative pain, and a shorter recovery period.^5,6^ As implant-based breast reconstruction continues to be the most common form of reconstruction, it is essential to explore prophylactic measures that can further mitigate the risk of wound healing complications in patients requiring radiotherapy.

One approach to reducing complications is the use of closed-incision negative pressure wound therapy (ciNPWT) systems such as Prevena (Incision Management System, Kinetic Concepts Inc., San Antonio, TX). CiNPWT is designed to protect surgical incisions from external contaminants, reduce edema, and promote perfusion and angiogenesis, thereby potentially reducing the incidence of complications.^7,8^ CiNPWT has been utilized in many surgical subspecialties to decrease surgical-site infection and wound-healing complications.^7,9,10^

The application of ciNPWT in the field of breast reconstruction has shown promising results in decreasing some of the most common complications associated with implant-based reconstruction, including reduced rates of mastectomy flap necrosis, dehiscence, infection, and seroma formation.^11-13^ However, there is currently a lack of focused research investigating the effectiveness of ciNPWT in reducing complications following breast reconstruction in patients who have undergone breast radiotherapy.

This study, therefore, is designed to fill this gap in the literature by examining the incidence of postoperative complications—specifically fluid collection, infection, and wound-healing complications—in breast reconstruction patients receiving ciNPWT treatment as opposed to those receiving standard dressings.

## METHODS

### Study Population

We undertook a retrospective single-center cohort study at our institution to investigate the effectiveness of ciNPWT in reducing postoperative complications among patients who underwent 2-stage implant-based breast reconstruction postmastectomy and radiotherapy. This study strictly adhered to ethical guidelines and secured approval from the IRB of our institution. The study encompassed consecutive patients between January 1, 2015, and December 31, 2022. Eligible participants were individuals aged 18 years or older who had previously undergone tissue expander placement or implant exchange and a subsequent follow-up spanning at least 6 months. Furthermore, all these patients had undergone radiation therapy a minimum of 6 months prior to their breast reconstruction. We excluded patients who denied Minnesota research authorization, patients with <6 months of follow-up, and patients who had <7 days of ciNPWT therapy due to device malfunction from air leak.

### Surgical Technique

All reconstructive surgeries were carried out by 1 of 4 experienced plastic surgeons from our institution, while the mastectomies were performed by 1 of 6 experienced breast surgical oncologists. The range of mastectomy procedures varied from skin-sparing to nipple-sparing mastectomies. To ensure uniformity in surgical approaches and mastectomy flap thickness, all surgeons rigorously followed a standardized protocol. The implants were placed in the prepectoral plane in all cases. All tissue expanders were meticulously wrapped with either Allergan’s AlloDerm SELECT Regenerative Tissue Matrix (16 × 20 cm size, 1.6 ± 0.4 mm thickness; Irvine, CA) or RTI Surgical’s Cortiva Allograft Dermis (16 × 20 cm size, 0.8-1.0 mm thickness; Alachua, FL). To mitigate infection risks, the surgical team adhered to stringent measures: gloves were replaced prior to every implant insertion, and the surgical site underwent irrigation using both antibiotic solutions and betadine. All tissue expanders were inflated within a range of 0 to 200 cc of air, depending on each case’s specifics, and surgical incisions were uniformly sutured in layers.

### Antibiotic Protocol and Drain Use

All patients were given antibiotics for the duration of the drains being present, which ranged from 7 to 14 days. The antibiotics administered were either cefadroxil 500 mg twice daily or clindamycin 300 mg every 6 h if the patient was allergic to cefadroxil. All tissue expander patients received one 15 Fr drain in each breast pocket that remained in until the output was <30 cc for 2 consecutive days. None of the implant patients had drains placed.

### Dressing

**Figure 1.**
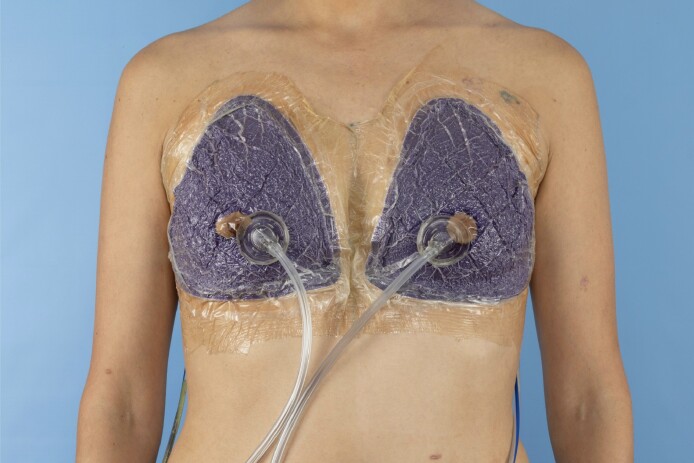
A photograph of the application of a closed-incisional negative pressure wound therapy on a 33-year-old female in immediate expander-based breast reconstruction.

Among the 4 plastic surgeons, 1 consistently utilized ciNPWT for every radiated breast under reconstruction. The other 3 surgeons opted for Steristrips complemented by brown paper tape. Patients undergoing ciNPWT had a tailored sponge dressing applied over the incision and adjacent soft tissue. This dressing was then secured firmly using adhesive tape. The negative pressure was maintained at 125 mmHg, continuously administered over a period of 7 days (Figure 1). All patients were discharged on the same day of surgery, including the ciNPWT cohort, which utilized the homegoing negative pressure device.

### Outcome Measures

Three primary postoperative complications were identified: fluid collection, infection, and wound-healing complications.

Fluid collection complications were identified as either hematoma or seroma and were only documented when there was a clinical need for aspiration. Infections were recognized either by purulent drainage from the surgical incision, a positive culture result obtained aseptically, or by the presence of clinical signs or symptoms, such as erythema, warmth, fever, pain, tenderness, and swelling.

Wound complications encompassed wound dehiscence or flap necrosis that mandated either dressing changes or more invasive surgical interventions. All patients in the study, with the exception of 2, were consistently followed up after their procedures. This follow-up continued until the onset of a complication, the event of death, or their final follow-up appointment, whichever came first.

### Statistical Analysis

Our analysis employed a procedure-level model, allowing patients undergoing bilateral or multiple surgeries to contribute a distinct observation for each procedure. Data for this study were meticulously collated, with categorical variables summarized using frequencies and percentages, and continuous variables represented through means and standard deviations (SDs).

### Propensity Score Weighting

Given the nonrandomized nature of the treatment assignment, there were inherent differences in baseline characteristics between the ciNPWT and non-ciNPWT groups. To adjust for potential confounding factors, propensity score methods were employed. The propensity score for each patient, which represents the probability of receiving ciNPWT based on observed covariates, was estimated using logistic regression. This model included variables such as BMI, smoking status, and diabetes mellitus (DM) status.

Following propensity score estimation, the inverse probability of treatment weights (IPTWs) was calculated. These weights balance observed covariates between the ciNPWT and non-ciNPWT groups, essentially creating a pseudo-population, where the distribution of observed baseline covariates is independent of treatment assignment. By utilizing IPTW, we were able to mitigate biases arising from confounding variables and achieve a comparison that closely resembles that of a randomized controlled trial.

With the weights applied, a weighted logistic regression was performed to compare the odds of each postoperative complication between the ciNPWT and non-ciNPWT groups. Odds ratios, along with their *P*-values, were reported to quantify the strength and significance of associations.

The datasets generated and analyzed during the current study are not publicly available due to privacy and ethical restrictions. However, de-identified data can be made available from the corresponding author upon reasonable request and in compliance with our institution’s data sharing policies and procedures.

## RESULTS

Our study evaluated a total of 315 breast reconstructions following radiotherapy (58 ciNPWT, 257 non-ciNPWT) performed between 2015 and 2022, with a total of 231 unique female patients. After excluding 7 patients who denied Minnesota research authorization, 2 patients with no postreconstruction follow-up, and 2 patients with premature removal of ciNPWT due to unrepairable air leak, we analyzed a final sample of 301 breasts (52 ciNPWT, 249 non-ciNPWT) among 218 unique patients.

Surgical characteristics were compared (Table 1). For the implant exchange procedure, 75.00% (39 out of 52) of the ciNPWT patients underwent the surgery, compared with 70.28% (175 out of 249) of the non-ciNPWT patients. Similarly, concerning the mastectomy with tissue expander placement procedure, 19.23% (10 out of 52) of the ciNPWT group underwent the surgery, compared with 29.32% (73 out of 249) of the non-ciNPWT group. Both variations were not statistically significant (*P* = .6069 and *P* = .1903, respectively). The mean duration from completion of radiation therapy to surgery was 5.37 years (SD 4.2) in the ciNPWT group and 5.12 years (SD 4.4) in the non-ciNPWT group (*P* = .682). Regarding the type of acellular dermal matrix used, there was no statistical significance between the use of Allergan’s AlloDerm SELECT (62.3%) and RTI Surgical’s Cortiva Allograft Dermis (37.7%; *P* = .824).

**Table 1.** Comparison of Surgical Procedures Between Breasts Treated With and Without ciNPWT

	ciNPWT			
	No (*n* = 249)	Yes (*n* = 52)	Total (*n* = 301)	*P*-value
Surgical procedures, *n* (%)				
Immediate tissue expander	74 (29.7)	13 (25.00)	87 (28.9)	.1903
Second-stage implant exchange	175 (70.28)	39 (75.00)	214 (71.09)	.6069

ciNPWT, closed-incision negative pressure wound therapy.

Demographic data (Table 2) revealed that the mean age at procedure was 50.7 years (SD 11.1), and the mean BMI was 27.2 (SD 5.2). The non-ciNPWT group had a slightly higher mean BMI (27.5 [SD 5.3]) compared with the ciNPWT group (25.9 [SD 4.5], *P* = .09). The non-ciNPWT group also displayed a higher smoking prevalence (30.1% vs 19.0%, *P* = .15; Table 2).

**Table 2.** Comparison of Demographics Between Patients Treated With and Without ciNPWT

	ciNPWT			
	No (*n* = 176)	Yes (*n* = 42)	Total (*n* = 218)	*P*-value
Age at procedure				.284^a^
*n*	176	42	218	
Mean (SD)	50.4 (10.98)	52.4 (11.69)	50.7 (11.13)	
BMI				.085^a^
*n*	176	42	218	
Mean (SD)	27.5 (5.33)	25.9 (4.45)	27.2 (5.19)	
BMI, *n* (%)				.427^b^
<18.5	2 (1.1)	1 (2.4)	3 (1.4)	
18.5-24.9	58 (33.0)	17 (40.5)	75 (34.4)	
25.0-29.9	66 (37.5)	16 (38.1)	82 (37.6)	
>30	50 (28.4)	8 (19.0)	58 (26.6)	
Smoking status, *n* (%)				.372^b^
No smoking history	123 (69.9)	34 (81.0)	157 (72.0)	
Former	51 (29.0)	8 (19.0)	59 (27.1)	
Active	2 (1.1)	0 (0.0)	2 (0.9)	
Ever smoked, *n* (%)				.151^c^
Never	123 (69.9)	34 (81.0)	157 (72.0)	
Ever	53 (30.1)	8 (19.0)	61 (28.0)	
Hypertension, *n* (%)				.302^c^
No	135 (76.7)	29 (69.0)	164 (75.2)	
Yes	41 (23.3)	13 (31.0)	54 (24.8)	
Diabetes mellitus, *n* (%)				1.000^b^
No	168 (95.5)	40 (95.2)	208 (95.4)	
Yes	8 (4.5)	2 (4.8)	10 (4.6)	
Dyslipidemia, *n* (%)				.829^c^
No	119 (67.6)	27 (65.9)	146 (67.3)	
Yes	57 (32.4)	14 (34.1)	71 (32.7)	
Missing	0	1	1	
CAD/PVD, *n* (%)				1.000^b^
No	172 (97.7)	41 (97.6)	213 (97.7)	
Yes	4 (2.3)	1 (2.4)	5 (2.3)	
DVT/PE, *n* (%)				.229^b^
No	169 (96.0)	38 (90.5)	207 (95.0)	
Yes	7 (4.0)	4 (9.5)	11 (5.0)	
Anticoagulants, *n* (%)				.247^b^
No	173 (98.3)	40 (95.2)	213 (97.7)	
Yes	3 (1.7)	2 (4.8)	5 (2.3)	
Steroids, *n* (%)				.578^b^
No	173 (98.3)	41 (97.6)	214 (98.2)	
Yes	3 (1.7)	1 (2.4)	4 (1.8)	

CAD/PVD, coronary artery disease or peripheral vascular disease; ciNPWT, closed-incision negative pressure wound therapy; DVT/PE, deep vein thrombosis or pulmonary embolism. ^a^Equal variance 2 sample *t* test. ^b^Fisher exact *P*-value. ^c^*χ*^2^*P*-value.

To ensure a fair comparison between the 2 groups, we employed IPTWs. These weights, derived from propensity scores, enabled us to adjust for potential confounding factors, including BMI, smoking status, and DM status. The distributions of other characteristics, such as hypertension, dyslipidemia, coronary artery disease or peripheral vascular disease, deep vein thrombosis or pulmonary embolism, and anticoagulant and steroid use were similarly balanced across ciNPWT status (all *P*-values >.2, Table 1).

During a mean postprocedural follow-up of 2.2 years (SD 1.6), the occurrence of fluid collection was observed in 2 patients (3.8%) in the ciNPWT group and 15 patients (6.0%) in the non-ciNPWT group, although the difference was not statistically significant (odds ratio: −0.0286, *P* = .23112). Infection was notably absent in the ciNPWT group, compared with 26 patients (10.4%) in the non-ciNPWT group (odds ratio: −0.1018, *P* < .0001). Lastly, the incidence of wound-healing complications was significantly lower in the ciNPWT group with only 1 case (1.9%), as opposed to 28 patients (11.2%) in the non-ciNPWT group (odds ratio: −0.0782, *P* = .00848; Table 3). Four patients experienced both fluid collection and infection, while none of the patients with wound-healing complications shared any other complications.

**Table 3.** Comparison of Postoperative Complications Between Patients Treated With and Without ciNPWT

	ciNPWT			
	No (*n* = 249)	Yes (*n* = 52)	Odds ratio	*P*-value
Complications				
Fluid collection	15	2	−0.0286	.23112
Infection	26	0	−0.1018	.00001
Wound healing	28	1	−0.0782	.00848

ciNPWT, closed-incision negative pressure wound therapy.

**Figure 2.**
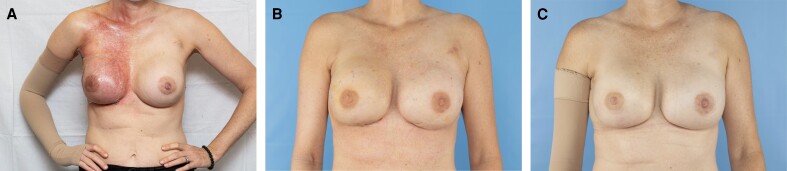
(A) Preoperative photograph of a 44-year-old female with a BMI of 22, history of bilateral nipple-sparing mastectomy with tissue expander placement followed by right breast radiation. (B) Postoperative appearance 10 days after implant exchange with closed-incision negative pressure wound therapy (ciNPWT) application. (C) Final appearance 1 year after implant exchange with ciNPWT application.

Highlighting the tangible impact of ciNPWT, Figure 2A-C visually chronicle the journey of a patient from just after radiation therapy, through 10 days postimplant exchange, to a year after the procedure.

## DISCUSSION

Our study aimed to investigate the efficacy of ciNPWT in reducing postoperative complications in a high-risk patient population undergoing breast reconstruction following mastectomy and radiotherapy. The data from 301 radiated breast reconstructions, 52 of which received ciNPWT treatment, suggest that the use of ciNPWT in this patient population could significantly reduce the incidence of common complications.

### Physiologic Mechanisms of Closed-Incision Negative Pressure Wound Therapy

CiNPWT^7,8^ works through several physiologic mechanisms that contribute to its efficacy in reducing postoperative complications, including infection, fluid accumulation, and delayed healing:

- Reduction of edema and hematoma formation: The application of negative pressure helps to reduce edema by continuously removing interstitial fluid from the wound site. This prevents the accumulation of fluid that can lead to hematoma and seroma formation. By managing fluid levels, ciNPWT minimizes the risk of fluid collections that can complicate healing and create an environment conducive to infection.
- Enhanced perfusion and angiogenesis: Negative pressure therapy promotes microcirculation within the wound bed, enhancing blood flow to the area. Improved perfusion delivers more oxygen and nutrients necessary for tissue repair and regeneration. Additionally, ciNPWT stimulates angiogenesis, the formation of new blood vessels, which further supports the healing process and strengthens the wound site against potential infections.
- Mechanical stabilization of the wound: The negative pressure exerted by ciNPWT stabilizes the wound edges, bringing them closer together and reducing the mechanical stress on the incision. This stabilization aids in faster and more effective wound closure, reducing the likelihood of dehiscence and other wound-healing complications.
- Reduction of bacterial load: By continuously evacuating exudate from the wound site, ciNPWT reduces the bacterial load and decreases the risk of infection. The sealed environment created by the therapy also serves as a barrier to external contaminants, further protecting the wound from bacterial invasion.
- Promotion of granulation tissue formation: CiNPWT encourages the formation of granulation tissue, which is essential for wound healing. Granulation tissue provides a foundation for epithelialization and wound closure, contributing to more robust and durable healing.

These physiologic mechanisms collectively contribute to the observed reductions in postoperative complications such as infection, fluid accumulation, and delayed healing in patients undergoing breast reconstruction with ciNPWT.

Of particular note, none of the procedures that used ciNPWT experienced postoperative infections (odds ratio: −0.1018, *P* < .0001). This finding suggests a strong preventive potential of ciNPWT against infections following breast reconstruction. This is a key observation, especially considering previous research showing that infection was one of the most frequent causes of hospital admission or surgical intervention in patients undergoing breast reconstruction after radiotherapy.^1^

The observed reductions in fluid collection and wound-healing complications further underscore the potential benefits of using ciNPWT in this patient cohort. Although the reduction in fluid collection complications did not reach statistical significance, the positive trend suggests a direction for future, larger studies that could elucidate this relationship further.

Although our study focused on the immediate postoperative healing phase, it is important to note that the long-term outcomes of ciNPWT use have not been extensively studied. Future research should investigate whether ciNPWT confers any long-term benefits, such as reduced capsular contracture rates. Capsular contracture, a common complication in implant-based breast reconstruction, significantly impacts patient outcomes and quality of life. Understanding the potential of ciNPWT to mitigate this complication could further solidify its role in breast reconstruction.

The rigorous methodology employed in this study, which included propensity score weighting and careful adjustment for potential confounding factors, strengthens the reliability of these findings. Moreover, the use of a procedure-level analysis model provided a nuanced view of patient outcomes, further enhancing the validity of our results.

### Device Limitations and Patient Experience

Although the benefits of ciNPWT are clear, there are several limitations and considerations associated with its use. Patient comfort with the device was generally well-reported, although the device requires some experience to place correctly to avoid air leaks. Air leaks were common, both in the postoperative setting and at home. In the postoperative area, significant reinforcement with dressings was sometimes required to maintain the seal. At home, air leaks sometimes occurred, necessitating either patient-guided patching over the phone using dressings provided at discharge or a visit to the hospital for assistance from the on-call physician.

Unrepairable air leaks, though not common, did occur in 2 patients and led to premature discontinuation of therapy, and were excluded from this study.

Although our study utilized the Prevena Bellaform system, we believe that the positive results observed with ciNPWT are not exclusive to this specific device. Similar outcomes could likely be achieved with other negative pressure wound therapy systems, provided they are applied and managed correctly.

Despite these promising findings, it is important to acknowledge the limitations of our study. The retrospective design may introduce selection bias, and the small sample size limits our statistical power to detect differences, particularly for less common complications. Additionally, while our propensity score weighting attempted to control confounding, the presence of unmeasured or residual confounding variables, such as the surgical technique of the surgeon using the ciNPWT or patient adherence to postoperative care instructions, cannot be ruled out. It should also be noted that the comparison of results from a single surgeon using ciNPWT may reflect individual surgical techniques rather than the efficacy of the ciNPWT alone. A comparison of results with and without the ciNPWT within the same surgeon’s practice would provide a more robust evaluation of the therapy's benefits.

## CONCLUSIONS

Our research contributes to the growing body of evidence, supporting the benefits of ciNPWT use in breast reconstruction by reducing wound-healing complications and infections, particularly in patients with a history of breast radiotherapy. These findings align with the broader scientific literature, which supports the use of ciNPWT systems in promoting surgical wound-healing and reducing complications. Future prospective studies with larger sample sizes are needed to confirm these findings and to assess the cost-effectiveness of ciNPWT in this context. Additionally, future research should focus on long-term outcomes, including the potential impact of ciNPWT on capsular contracture rates, to provide a comprehensive understanding of its benefits in breast reconstruction.
